# Identification of *KIF18B* as a Hub Candidate Gene in the Metastasis of Clear Cell Renal Cell Carcinoma by Weighted Gene Co-expression Network Analysis

**DOI:** 10.3389/fgene.2020.00905

**Published:** 2020-08-20

**Authors:** Huiying Yang, Yukun Wang, Ziyi Zhang, Hua Li

**Affiliations:** ^1^Department of Nephrology, Sir Run Run Shaw Hospital, Zhejiang University School of Medicine, Hangzhou, China; ^2^Department of Urology, Sir Run Run Shaw Hospital, Zhejiang University School of Medicine, Hangzhou, China; ^3^Department of Endocrinology, Sir Run Run Shaw Hospital, Zhejiang University School of Medicine, Hangzhou, China

**Keywords:** clear cell renal cell carcinoma, weighted gene co-expression network analysis, enrichment analysis, maximal clique centrality, survival analysis, precise therapies, sing-gene gene set enrichment analysis

## Abstract

**Background:**

Clear cell renal cell carcinoma (ccRCC) is a common type of fatal malignancy in the urinary system. As the therapeutic strategies of ccRCC are severely limited at present, the prognosis of patients with metastatic carcinoma is usually not promising. Revealing the pathogenesis and identifying hub candidate genes for prognosis prediction and precise treatment are urgently needed in metastatic ccRCC.

**Methods:**

In the present study, we conducted a series of bioinformatics studies with the gene expression profiles of ccRCC samples from Gene Expression Omnibus (GEO) and the cancer genome atlas (TCGA) database for identifying and validating the hub gene of metastatic ccRCC. We constructed a co-expression network, divided genes into co-expression modules, and identified ccRCC-related modules by weighted gene co-expression network analysis (WGCNA) with data from GEO. Then, we investigated the functions of genes in the ccRCC-related modules by enrichment analyses and built a sub-network accordingly. A hub candidate gene of the metastatic ccRCC was identified by maximal clique centrality (MCC) method. We validate the hub gene by differentially expressed gene analysis, overall survival analysis, and correlation analysis with clinical traits with the external dataset (TCGA). Finally, we explored the function of the hub gene by correlation analysis with targets of precise therapies and single-gene gene set enrichment analysis.

**Results:**

We conducted WGCNA with the expression profiles of GSE73731 from GEO and divided all genes into 8 meaningful co-expression modules. One module is proved to be positively correlated with pathological stage and tumor grade of ccRCC. Genes in the ccRCC-related module were mainly enriched in functions of mitotic cell division and several proverbial tumor related signal pathways. We then identified *KIF18B* as a hub gene of the metastasis of ccRCC. Validating analyses in external dataset observed the up-regulation of *KIF18B* in ccRCC and its correlation with worse outcomes. Further analyses found that the expression of KIF18B is related to that of targets of precise therapies.

**Conclusion:**

Our study proposed *KIF18B* as a hub candidate gene of ccRCC for the first time. Our conclusion may provide a brand-new clue for prognosis evaluating and precise treatment for ccRCC in the future.

## Introduction

Renal cell carcinoma (RCC) is one of the top 10 prevalent malignancies and makes up approximately 2–3% of all cancers (Ljungberg et al., 2015). Clear cell renal cell carcinoma (ccRCC) is the most familiar histological subtype of RCC (Ochocki et al., 2018), the pathogenesis of which is still far from clear. As approximately one third of ccRCC patients were diagnosed with distant metastasis (Gupta et al., 2008) and the disease has low insensitivity toward traditional chemotherapy or radiotherapy, metastasis accounts for about 90% of ccRCC-related mortality (Chaffer and Weinberg, 2011). Nonetheless, precise treatments, such as targeted therapy (Campbell et al., 2018) and immunological therapy (Albiges et al., 2019), have shown relatively satisfactory effects in the treatment of metastatic ccRCC. Hence, it has become an urgent mission to identify novel hub candidate genes behind the mechanism of the metastasis, which may provide valuable targets for precise therapies.

At present, the pathogenesis of ccRCC has been partially clarified. The complete loss mutation through genetic and/or epigenetic mechanisms of the von Hippel-Lindau (VHL) tumor suppressor gene is regarded as the earliest and most significant oncogenic factor in ccRCC. The loss mutation of VHL leads to aberrant accumulation of hypoxia-inducible factors (HIF) even if the tissue microenvironment is adequately oxygenated, which results in abnormal activation of HIF targeting genes and then regulates the processes of angiogenesis, glycolysis, and apoptosis. The genetic diversity in ccRCC provides the substrate in ccRCC, and the selection upon the substrate enables the tumor to adapt to pressures and metabolic demands. Except for the mechanism in genetic field, analyses of gene expression, metabolic, and immunological status of ccRCC have given important mechanistic and clinical insights in ccRCC as well (Hsieh et al., 2017).

Thanks to contemporary breakthroughs of biological techniques, bioinformatics analyses have become new approaches for uncovering the pathogenesis of diseases. Besides studies about genetic sequence and mutations, researches focusing on gene expression levels have attracted more attention. Among various means for expression profile analysis, weighted gene co-expression network analysis (WGCNA) (Langfelder and Horvath, 2008) stands out because of its superiorities in identifying hub candidate genes involved in diseases. WGCNA could divide genes with similar expression patterns into several biologically meaningful co-expression modules, analyze the relationship between gene modules and clinical traits, and finally evaluate the significance of genes in trait-related modules and excavate the hub candidate genes underlying the mechanism of diseases.

WGCNA have been widely used in various medical fields, such as tumor (Xiang Z. et al., 2019), neurological and psychiatric disorders (Katrinli et al., 2019; Tang and Liu, 2019), chronic disease (Chen et al., 2019), and infectious diseases (Bando et al., 2019). What’s more, most of the conclusions drawn from WGCNA can be further confirmed by bioinformatics analyses or biological experiments, which guarantees the high reliability of WGCNA. WGCNA has been used for screening hub genes in ccRCC as well, and more efforts are needed for exploring novel hub genes blamed for metastasis or could act as potential targets for precise treatment.

Our study constructed a weighted co-expression network with the expression profiles of ccRCC tissues and related co-expression modules with clinical traits. Then we analyzed the main functions of genes in the trait-related module by enrichment analyses and successfully identified a hub candidate gene of ccRCC. Finally, we validated the reliability and clinical significance of the hub gene and explored its functions with an external dataset. We expect that our study could make a contribution to explain the pathogenesis of the metastasis of ccRCC and provide a potential target for treatment.

## Materials and Methods

### Data Collection and Pre-processing

**FIGURE 1:**
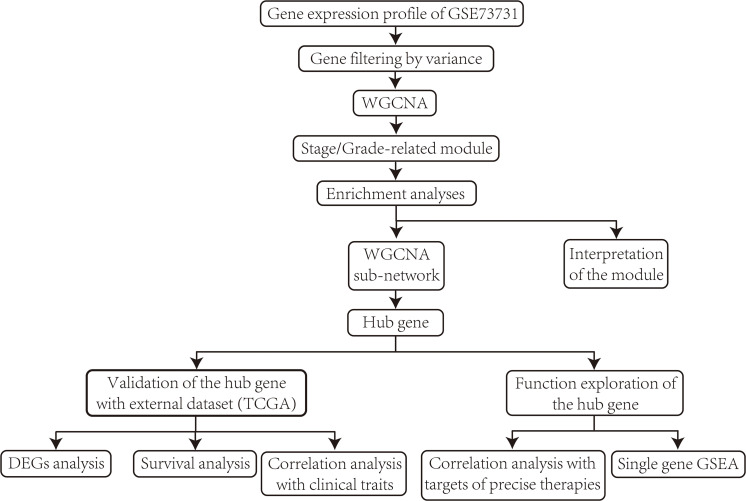
Flow chart of data collection, data pre-processing, data analyzing, identification, validation, and function exploration of hub gene.

The overall design and procedures are described in a flow chart (Figure 1).

We searched Gene Expression Omnibus (GEO)^1^ database with the keyword “clear cell renal cell carcinoma” and decided on dataset GSE73731 (Wei et al., 2017) for hub gene extracting as it has relative large sample size and detailed clinical information (gender, pathological stage, and tumor grade included). Samples without information of clinical traits were excluded from analyses containing clinical information (details of samples with complete clinical information are available in Supplementary Table S1). After downloading the raw data (already log2 transformed), we carried out probe annotation with microarray platform file under R environment. Probes matching more than one gene were discarded, and average values were taken for genes detected by more than one probes. Before WGCNA, we conducted sample clustering with hierarchical clustering method and excluded outlier samples accordingly. We filtered non-varying genes in the whole expression profile by variance as they are deemed as noise and may result in adverse effects to WGCNA.

For the validation of hub gene, we obtained the expression profile of ccRCC from the database of the cancer genome atlas (TCGA)^2^. TCGA included the expression profile and clinical information of 72 normal control and 539 ccRCC patients.

### Construction of Weighted Co-expression Network and Division of Co-expression Modules

We conducted WGCNA with the WGCNA package (Langfelder and Horvath, 2008) under R environment.

Firstly, we constructed a Pearson’s correlation matrix of all pairwise genes by Pearson’s correlation analysis. Secondly, we converted the Pearson’s correlation matrix into an adjacency matrix (scale-free network) by a β the power operation (β value was known as the soft-thresholding value). To decide the most appropriate β value, we calculated the scale-free fit index and mean connectivity for each supposed β from 1 to 20. As higher scale-free fit index represents better coincidence with scale-free network and higher mean connectivity means better connection of the whole network, we referred to both of the indexes and decided the β value with scale-free fit index bigger than 0.85 as well as highest mean connectivity as the proper one. Then, we transformed the adjacency matrix into a topological overlap matrix (TOM) by calculating the topological overlap between pairwise genes, by which we could take indirect correlations into consideration as well as reduce noise and spurious correlations. Finally, we used the average linkage hierarchical clustering based on the TOM-based dissimilarity measure to divide genes into several co-expression modules, so that genes with co-expression relationships were gathered in the same module and genes expressed separately were divided. Modules of high similarity (higher than 0.75) were merged together.

### Identification of Clinically Meaningful Modules

The clinical traits of GSE73731 contain gender, pathological stage, and tumor grade. To excavate hub genes related with the advancement and metastasis of ccRCC, we mainly aimed at modules of positive correlation with the trait of stage and grade. We conducted module-trait correlation analysis by Spearman’s correlation analysis between module eigengene (ME, the first principal component of a given module) and clinical traits (stages I–IV was represented as 1, 2, 3, 4, and so do G1–G4). Modules of significant correlations with traits of pathological stage or tumor grade are defined as ccRCC-related modules, and genes in such modules were extracted for subsequent hub gene extraction.

We introduce the conceptions of Gene significance (GS) and Module Membership (MM) (Langfelder and Horvath, 2008) here. GS represents the correlation of the expression level of a gene and a clinical trait, and MM means the Pearson’s correlation of the expression level of a certain gene and the module eigengene. An ideal clinic-related module is supposed to contain genes of high correlation between GS and MM.

### Enrichment Analyses on ccRCC-Related Module

Gene Ontology (GO) (Lu et al., 2008) enrichment analysis and Kyoto Encyclopedia of Genes and Genomes (KEGG) (Ogata et al., 1999) pathway enrichment analysis could reveal the biological processes and signal pathways in which certain gene cluster is involved. We performed enrichment analyses on genes in ccRCC-related modules and displayed the results with the clusterProfiler package (Yu et al., 2012) under R environment. The criteria for enriched terms were set as *p* < 0.01 and Benjamin-Hochberg adjusted *p* < 0.05. We mainly focus on the category of biological process (BP) among the results of GO enrichment analysis.

### Identification of Hub Gene

After GO enrichment analysis on trait-related module, we extracted the genes from the most statistically significant GO term and built a sub-network with the weighted correlations among them. Then, we employed Maximal Clique Centrality (MCC) with cytohubba (Chin et al., 2014), a plug-in of cytoscape (Shannon et al., 2003), to assess the centrality of each gene in the sub-network. Genes with top MCC values are deemed as potential hub candidate genes related with the metastasis of ccRCC.

### Validation of Hub Gene With Differentially Expressed Gene Analysis

To verify whether the hub gene is significantly up-regulated or down-regulated in ccRCC compared with normal control, we conducted differentially expressed gene (DEG) analysis on decided hub gene by Wilcoxon test method with limma package under R environment (Ritchie et al., 2015). DEGs analysis was conducted with the data obtained from TCGA. The cut-off criteria of DEGs were set as *p* < 0.01 and |logFC| > 0.5.

### Validation of Hub Gene With Survival Analysis

For validating whether the hub gene could affect the survival of ccRCC patients, we conducted overall survival (OS) analysis on the data obtained from TCGA with the survival package under R environment. Data from TCGA contains the expression profiles and follow-up information of 539 ccRCC samples. Samples with follow-up time less than 90 days were excluded. All samples were divided into two groups of high-expression or low-expression depending on the expression level of the hub gene. Then we conducted OS analysis with Kaplan-Meier method with a two-sided log-rank test to explore the difference of OS between the two groups.

### Validation of Hub Gene by Analyzing the Relationship Between the Expression Level of Hub Gene and Clinical Traits

We divided all ccRCC samples in TCGA into high-expression and low-expression group by the median of the expression level of the hub gene. Then, we conducted correlation analysis between the expression groups and clinical traits (age, gender, tumor grade, pathological stage, T stage, and distant metastasis) to confirm the validity of our hub gene. The analysis was conducted by chi-square test under R environment. The criterion for statistical significance was set as *p* < 0.01.

### Exploration of the Correlation Relationships Between the Expression Level of Hub Gene and Targets for Precise Therapies

Precise treatment, including immunotherapy and targeted therapy, is a novel approach for treating patients without opportunities for surgery or acting as a supplementary treatment before/after surgeries. Recently, more and more precise therapies were admitted for treating ccRCC by the Food and Drug Administration (FDA) (Barata and Rini, 2017). We analyzed the correlation relationships between the expression level of our hub gene and the targets of precise treatments by Pearson’s correlation analysis to validate the significance of the hub gene in ccRCC and estimate the potential capacity of predicting the therapeutic effects with our hub gene. The targets of precise therapies are listed as follows: programmed cell death 1 (*PD1*), programmed cell death ligand 1 (*PDL1*), vascular Endothelial Growth Factor Receptor (*VEGFR1*), Fms-like tyrosine kinase 3 (*FLT3*), vascular Endothelial Growth Factor Receptor 3 (*VEGFR3*), mammalian target of rapamycin (*mTOR*), platelet-derived growth factor receptor alpha (*PDGFRA*), platelet-derived growth factor receptor beta (*PDGFRB*), *KIT* proto-oncogene (*KIT*), ret proto-oncogene (*RET*), and *MET* protooncogene (*MET*).

### Function Analysis of the Hub Gene by Single-Gene Gene Set Enrichment Analysis

For exploring the biological function of hub gene in ccRCC, we conducted single-gene gene set enrichment analysis (GSEA) (Subramanian et al., 2005) on the hub gene with the data from TCGA. All samples were divided into high-expression and low-expression groups by the median of the hub gene, and GSEA was conducted to explore the up-regulated and down-regulated signal pathways in different groups. The criteria for statistical significance were set as *p* < 0.01 and *FDR* < 0.25.

## Results

### Data Pre-processing

**FIGURE 2:**
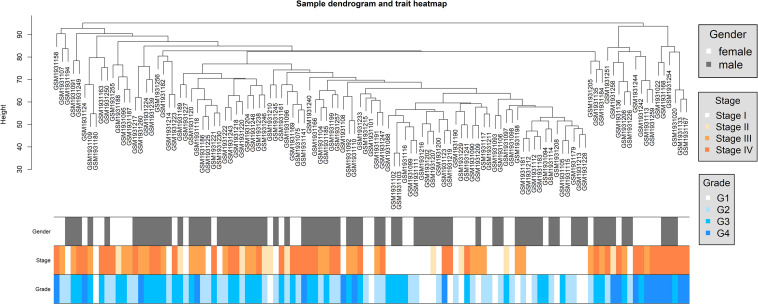
Dendrogram of sample clustering and heatmap of clinical traits of all ccRCC samples in dataset of GSE73731.

For GSE73731, we got an expression profile of 22,320 genes after probe annotation and reserved 113 ccRCC samples after sample clustering and outlier sample exclusion; 5,580 genes were adopted after gene filtering. The sample clustering tree depicted a satisfying result (Figure 2). Samples of similar pathological stages or tumor grades were gathered together, and the distance between samples with higher pathological stages or tumor grades and samples with lower pathological stages or tumor grades were relatively far.

### Weighted Gene Co-expression Network Construction and Module Division

**FIGURE 3:**
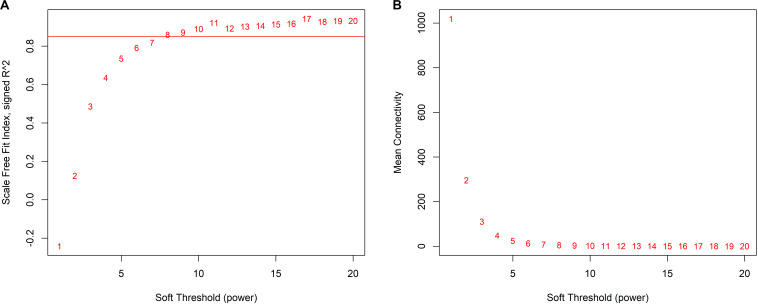
Determination of appropriate soft-thresholding values (β). **(A)** The scale-free fit index of supposed β value from 1 to 20. **(B)** The mean connectivity of supposed β value from 1 to 20.

**FIGURE 4:**
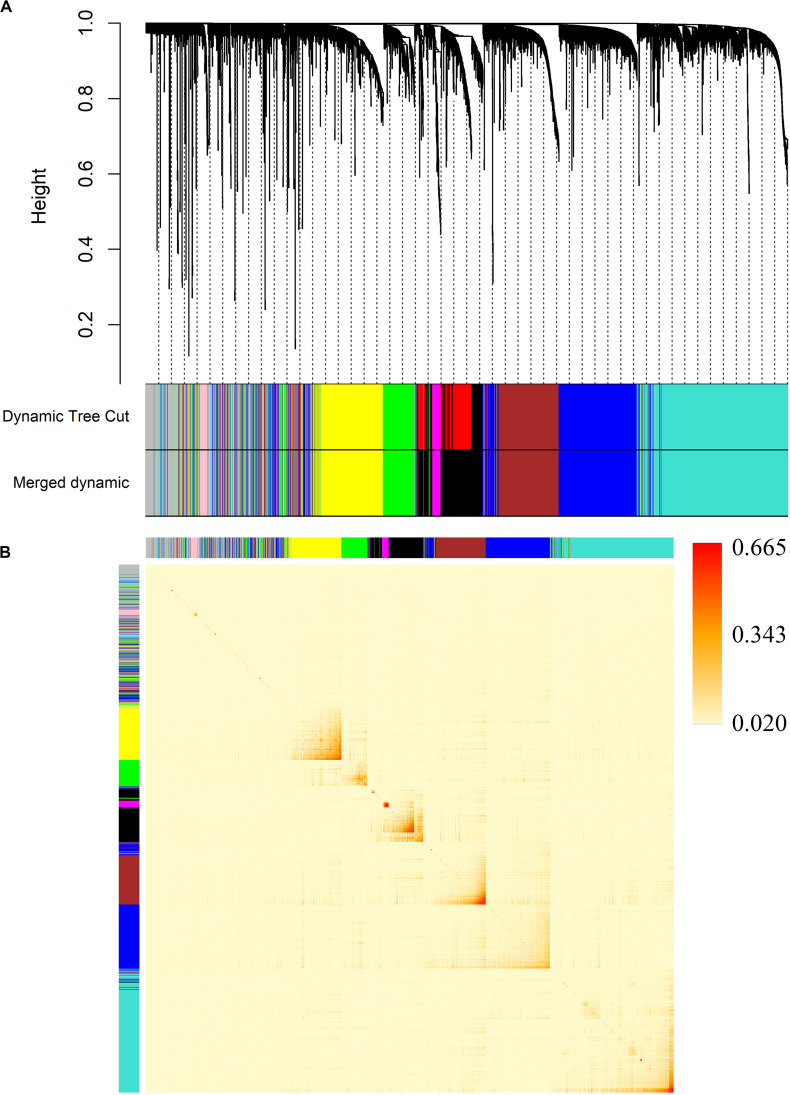
Division of co-expression gene modules and an adjacency heatmap of genes. **(A)** Dendrogram of all 5,580 genes clustered by average linkage hierarchical clustering based on the TOM-based dissimilarity measure. **(B)** The adjacency heatmap of 5,580 genes. The color intensity represents the weighted correlation coefficients between pairwise genes.

We decided 8 as the proper β value (Figure 3) and converted the expression matrix into a topological overlap matrix according to the method mentioned before. Then, 9 co-expression modules were divided from all 5,580 genes and distinguished with colors (Figure 4A). The brown, black, magenta, blue, turquoise, pink, green, yellow, and gray modules contained 711, 534, 77, 1,037, 1,394, 82, 408, 672, and 665 genes, respectively. The gray module contained genes that couldn’t be divided into any co-expression modules (all modules and corresponding genes are available in Supplementary Table S2).

In order to verify the accuracy of the module division, we mapped an adjacency heatmap of all analyzed genes (Figure 4B). The results indicated that genes showed stronger co-expression relationships with genes from the same module and weaker relationships with genes in other modules, which proved the preciseness of the module division.

### Module-Trait Relationship Analysis and Identification of ccRCC-Related Module

**FIGURE 5:**
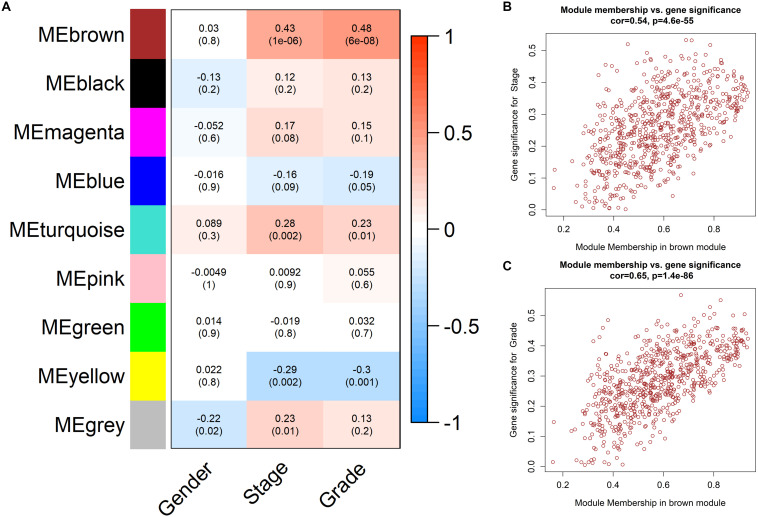
Determination of the ccRCC-related module. **(A)** Heatmap of trait-module correlations. The color of each cell reflects the correlation coefficients (red for positive correlation and blue for negative correlation). The statistical significances of correlation relationships were noted in the cells. The brown module was thus identified as a ccRCC-related module. **(B)** Scatter plot displaying correlation between Gene significance (GS) and Module Membership (MM) of pathological stage of genes in the brown module. **(C)** Scatter plot displaying correlation between Gene significance (GS) and Module Membership (MM) of tumor grade of genes in the brown module.

We calculated the correlation coefficients and corresponding statistical significance between module eigengenes and clinical traits and showed the results with a heatmap (Figure 5A). We found that the brown module was positively related to clinical trait of pathological stage and tumor grade of ccRCC simultaneously. That is to say, the up-regulation of genes in the brown module may have grate efforts on the metastasis of ccRCC. We renamed the brown module as the ccRCC-related module.

Further, we evaluated the correlation of GS and MM in the ccRCC-related module to find high correlation coefficients (cor = 0.54, *p* = 4.6E-55 and cor = 0.65, *p* = 1.4E-86, Figures 5B,C), so that the ccRCC-related module was regarded as an appropriate module for subsequent analyses and hub gene extraction.

### GO and KEGG Pathway Enrichment Analyses of the ccRCC-Related Module

**FIGURE 6:**
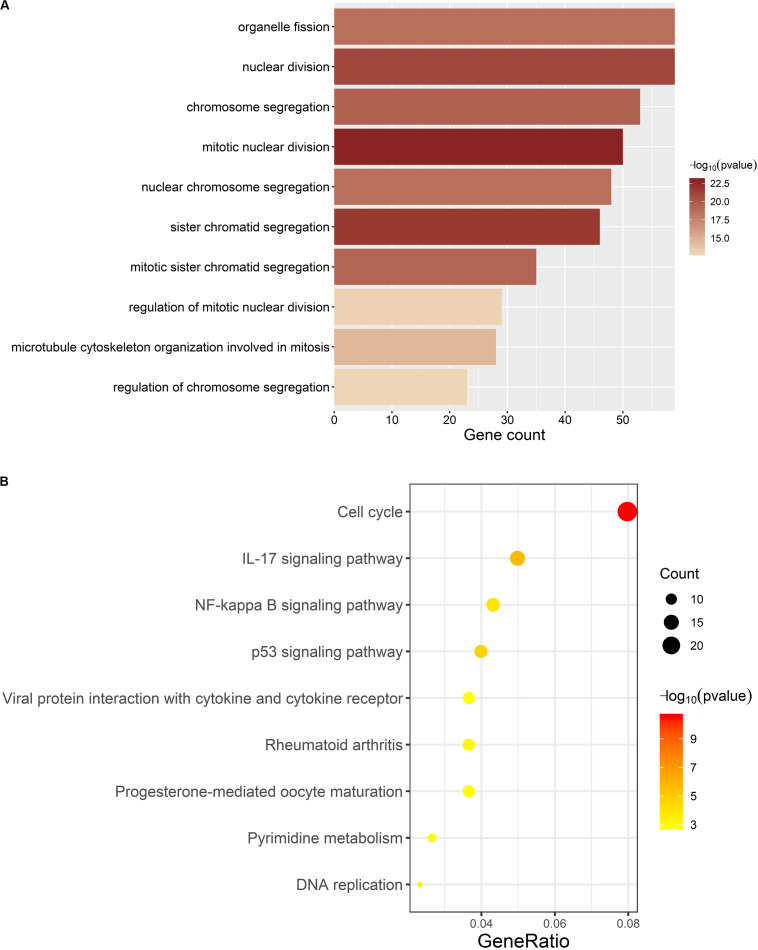
Results of enrichment analyses. **(A)** Top 10 GO-BP terms. The length and color of barplot displays the enriched gene count and the statistical significance for each term respectively. **(B)** All KEGG terms. The size and color of the dot represents the enriched gene count and statistical significance for each term, respectively. Gene ratio means the ratio of enriched gene count and all genes involved in KEGG enrichment analysis.

We carried out GO and KEGG pathway enrichment analyses on genes in ccRCC-related module to find out the mainly enriched biological processes and signal pathways. Figure 6 represents the top 10 terms of GO-BP and KEGG enrichment analyses (all enriched terms and the interpretations of the top-10 terms are available in Supplementary Table S3).

For GO-BP enrichment analysis (Figure 6A), the majority of the terms were about the procedure of mitotic cell division, such as “mitotic nuclear division” (gene count = 50, *p* = 1.11E-23), “sister chromatid segregation” (gene count = 46, *p* = 1.60E-22), and “nuclear division” (gene count = 59, *p* = 8.46E-22).

Results of KEGG pathway enrichment analysis (Figure 6B) were similar to that of GO-BP enrichment analysis. Genes in the brown module were mainly enriched in signal pathways about cell division, such as “Cell cycle” (gene count = 24, *p* = 2.82E-11) and “DNA replication” (gene count = 7, *p* = 0.0003), which are directly connected with the excessive proliferation of cells in tumor. Meanwhile, several items have already been proved to participate in the occurrence and development of multiple tumors, such as “IL-17 signaling pathway” (gene count = 15, *p* = 1.90E-06), “p53 signaling pathway” (gene count = 12, *p* = 1.46E-05), and “NF-kappa B signaling pathway” (gene count = 13, *p* = 0.0001).

### Excavation of the Hub Gene

**FIGURE 7:**
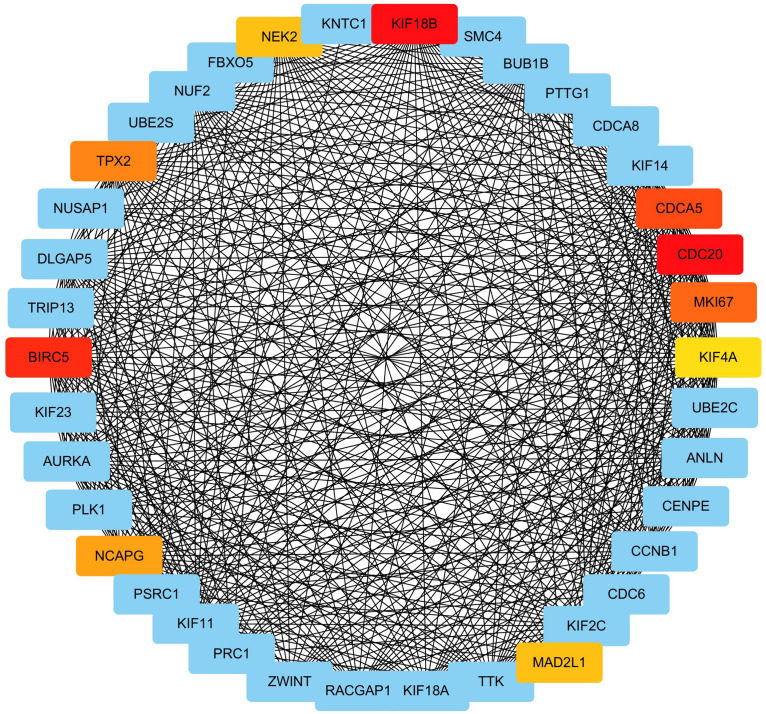
Identification of hub gene by MCC method. The MCC values of genes in the GO term “mitotic nuclear division” were calculated. Genes with top 10 MCC values were colored with red and yellow color and other genes was colored with blue. Among genes with top-10 MCC values, red means relative bigger MCC value and yellow means relative smaller MCC values, and the same color means the same MCC values.

We extracted genes as well as their weighted co-expression coefficients from the GO term of “mitotic nuclear division” (the most significantly enriched GO-BP term) and constructed a sub-network of the co-expression network. With the weighted correlations among genes, we analyzed the centrality of genes in the sub-network with MCC method (only top 500 correlations were concerned, Supplementary Table S4). Genes with higher MCC values were regarded as connecting more closely with others and playing crucial roles in the co-expression relationship. The results are displayed in Figure 7 and the top 10 central genes are highlighted.

The top 10 genes were *KIF18B*, *BIRC5*, *CDC20*, *CDCA5*, *MKI67*, *TPX2*, *NCAPG*, *MAD2L1*, *NEK2*, and *KIF4A*. *KIF18B* had the highest MCC value and is deemed as the hub gene of ccRCC as a result.

### Validation of KIF18B by Differentially Expressed Gene Analysis

**FIGURE 8:**
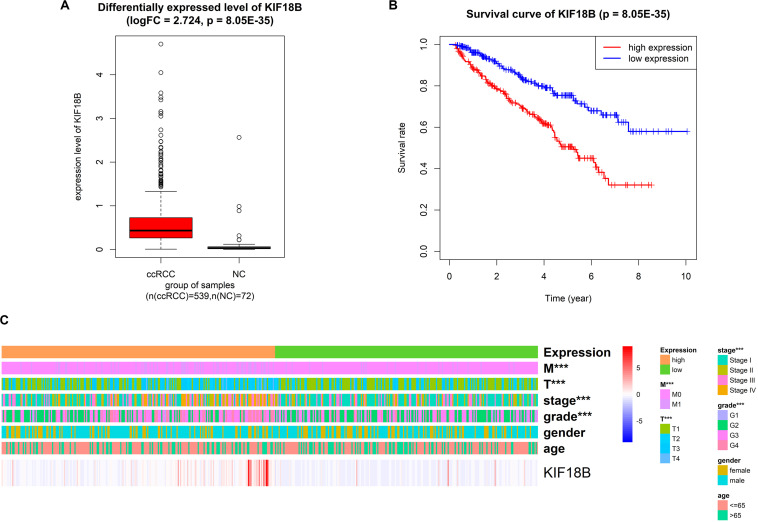
Validation of the reliability of *KIF18B* with external dataset (TCGA). **(A)** Differentially expressed gene analysis of *KIF18B* between ccRCC and normal control. **(B)** Overall survival analysis on *KIF18B*. **(C)** Correlation analysis between expression level of *KIF18B* and clinical traits (***p < 0.001).

We explored the differentially expressed level of *KIF18B* between ccRCC and normal control. The results illustrated that *KIF18B* was significantly up-regulated in ccRCC compared with control (logFC = 2.724, *p* = 8.05E-35, Figure 8A).

### Validation of KIF18B by Survival Analysis

We validated by survival analysis that ccRCC patients with higher expression level of *KIF18B* would have obviously worse prognostic outcomes and shorter overall survival. The 5-year survival rate of the high-expression group and the low-expression group was 50 and 75%, respectively (*p* = 9.48E-7, Figure 8B).

### Exploration of the Relationship Between the Expression of KIF18B and Clinical Traits

We analyzed the correlation between different expression group of *KIF18B* and clinical traits by chi-square test. The results verified that different expression group of *KIF18B* is significantly related to tumor grade (*p* = 2.43E-05), pathological stage (*p* = 6.394E-06), T stage (*p* = 3.178E-06), and distant metastasis (*p* = 2.043E-05), but it isn’t related to gender and age (Figure 8C; results of chi-square test are given in Supplementary Table S5).

### Correlation Analysis Between the Expression of KIF18B and Targets of Precise Treatments

**FIGURE 9:**
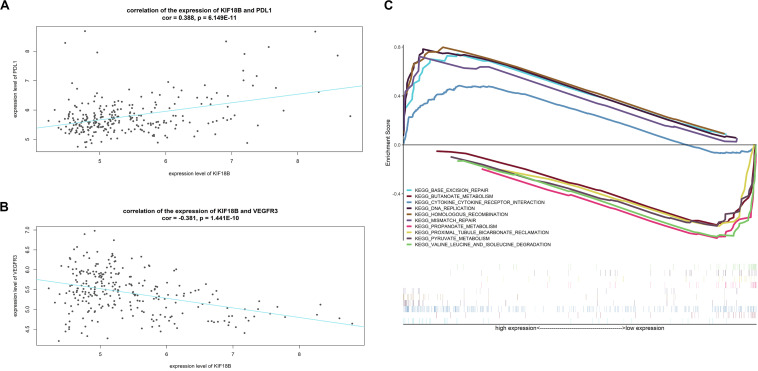
Function exploration of *KIF18B*. **(A)** Correlation analysis between the expression level of *KIF18B* and *PDL1*. **(B)** Correlation analysis between the expression level of *KIF18B* and *VEGFR3*. **(C)** Single-gene GSEA analysis of *KIF18B*.

The results of correlation analysis showed that the expression level of *KIF18B* is positively related to the expression level of *PDL1* (cor = 0.3788, *p* = 6.149E-11, Figure 9A) and is negatively related to the expression level of *VEGFR3* (cor = −0.381, *p* = 1.441E−10, Figure 9B), which hints that patients with high-expression of *KIF18B* might respond better to treatments targeting *PDL1* and worse to treatments targeting *VEGFR3*.

### Single-Gene GSEA on KIF18B

Figure 9C exhibits the top-5 up-regulated and top-5 down-regulated signal pathways in the high-expression group of *KIF18B* (all significant enriched terms are given in Supplementary Table S6). The results indicated that *KIF18B* is involved in the biological processes of “Base excision repair,” “DNA replication,” “Mismatch repair,” and “homologous recombination” in ccRCC.

## Discussion

In the current study, we selected the gene expression profile of GSE73731 from GEO database for conducting WGCNA and extracting the hub gene. For data processing, we removed the outlier samples after sample clustering and filtered genes of low variance. We conducted WGCNA and divided all genes into 8 meaningful co-expression modules. After trait-module correlation analysis, the brown module was identified as a key module of positive correlation with higher pathological stage and tumor grade in ccRCC, which means that genes in this module were related with the development and metastasis of ccRCC. Subsequent GO and KEGG enrichment analyses indicated that genes in the ccRCC-related module were mainly enriched in biological function of cell cycle, cell proliferation, tumor metastasis, and material metabolism, which agreed with the characteristics of tumor well. To screen out the hub gene of ccRCC from the ccRCC-related module, we pulled out the genes in the top GO-BP term and constructed a sub-network of WGCNA. Then, *KIF18B* was confirmed as the hub candidate gene with MCC method. We then validated the reliability of *KIF18B* with the external dataset obtained from TCGA database. DEGs analysis found that *KIF18B* was up-regulated in ccRCC patients compared with normal control and OS analysis revealed a worse prognostic outcome in patients with high expression level of *KIF18B*. Correlation analysis between the expression of KIF18B and clinical traits proved that the expression of *KIF18B* is significantly related to tumor grade, pathological stage, T stage, and distant metastasis. Finally, in order to explore the function and clinical significance of *KIF18B*, we analyzed the correlation between the expression level of *KIF18B* and targets of precise therapies and found the expression level of *KIF18B* is correlated with that of *PDL1* and *VEGFR3.* Meanwhile, single-gene GSEA revealed that *KIF18B* is mainly involved in DNA replication and mutation in ccRCC.

WGCNA is a powerful bioinformatics tool for extracting hub genes participating in the pathogenesis and affecting the prognosis of tumor. Most of the top 10 hub genes identified by MCC method in our study have already been proved as hub candidate genes of ccRCC. Numerous studies agreed that the overexpression of *BIRC5* means more advanced pathological stage, severer metastasis, and shortened overall survival (Pu et al., 2017). Studies concerning the effects of *CDC20* (Yuan et al., 2017), *KIF4A* (Wei et al., 2019), *NEK2* (Arai et al., 2015), *TPX2* (Wei et al., 2019), and *NCAPG* (Wei et al., 2019) on ccRCC have drawn similar conclusions. Most importantly, the overexpression of *MKI67* is closely related with the overall survival, pathological stage, and Fuhrman grade in ccRCC, so that it is regarded as a biomarker for the disease (Xie et al., 2017). As *KIF18B* was identified as a hub gene together with the above-mentioned genes by MCC method and WGCNA showed apparent co-expression relationships among *KIF18B* and these hub genes, the conclusions propose by other researchers demonstrate the high credibility of our results indirectly. After identification of *KIF18B*, we validated it with an external dataset obtained from TCGA to confirm the reliability of our conclusion. Finally, we explored the functions and the relationship of *KIF18B* with targets of precise therapies to discover the clinical value of our hub gene. In brief, *KIF18B* is worthy of deeper research as a hub candidate gene in ccRCC.

*KIF18B* is a protein-coding gene that encodes kinesin family member 18B, which is a member of over 40 different kinds of kinesin proteins (Hirokawa et al., 2009). Kinesin functions with dynein as motor proteins to carry out microtubule-regulated movement in many vital biological processes such as cell division and cargo transport (Stout et al., 2011; Wu et al., 2006). *KIF18B* mainly locates in the nucleus and its expression is cell cycle-dependent. Researches have shown that the expression level of *KIF18B* is remarkably elevated at late Second-Gap/Metaphase in cell cycles, demonstrating that *KIF18B* may act as an important mitosis-regulating motor protein (Lee et al., 2010). Studies have shown that *KIF18B* plays an important role during the process of Metaphase by regulating the movement of chromosomes from the spindle poles toward the spindle equator (Rath and Kozielski, 2012).

As it hasn’t been long since researchers noticed the importance of *KIF18B* for maintaining normal biological functions, there are relatively few studies about the relation between the disordered expression of *KIF18B* and diseases. Yet, several studies have shown close connections between *KIF18B* and cancer.

Wu et al. (2018) observed significant up-regulation of *KIF18B* in cervical cancer compared with normal control, and the up-regulation is positive correlated with the size of the primary tumor and tumor grade. The invasion capacities of the tumor cells were weakened after the knockdown of *KIF18B* by siRNAs *in vitro*. On the contrary, the overexpression of *KIF18B* promotes the proliferation, invasion, and migration of cancer cells. They hypothesized that *KIF18B* may function through *Wnt/*β-catenin pathway and verified the down-regulation of *C-myc*, β-catenin, and phosphorylated *GSK3*β after the knockdown of *KIF18B*. Moreover, the volumes and weight of the tumor were obviously reduced in mouse model after down-regulation treatment of *KIF18B*. The results have uncovered that *KIF18B* acts as a potential oncogene in cervical cancer.

Another research (Xiang X. H. et al., 2019) reveals that *KIF18B* is down-regulated in senescent cells and abnormally up-regulated in hepatocellular carcinoma cells, and the overexpression of *KIF18B* was a risk factor for poorer survival. Other reports have drawn similar conclusions in lung adenocarcinoma (Zhang et al., 2019) and bladder cancer (Pan et al., 2019). Accordingly, we suppose the high utilization value of *KIF18B* as a biomarker for prognosis evaluating and a specific target for precise treatment in ccRCC.

At the end of the article, we’d like to enumerate several limitations and future directions of our research. Firstly, the whole study was carried out on the basis of public databases (GEO and TCGA), so that we are attempting to collect some samples by ourselves and validate the results in more external datasets. Secondly, we will attempt to explore the effect of *KIF18B* on its related genes by analyzing the receptor-ligand relationships of *KIF18B* by further analyses of single-cell RNA-seq. Finally, the function of *KIF18B* should be further validated by not only bioinformatics analyses but also experiments in cells or animals. We’ll devote ourselves to conduct those studies to make our conclusions more complete.

## Conclusion

In summary, our study identified and validated *KIF18B* as a hub candidate gene of ccRCC by WGCNA and a series of systematic bioinformatics analyses. *KIF18B* may act as a potential biomarker for prognosis prediction, precise treatment in the future. Our conclusions provided novel insights for uncovering the mechanism of ccRCC.

## Data Availability Statement

Publicly available datasets were analyzed in this study. This data can be found here: https://www.ncbi.nlm.nih.gov/geo/query/acc.cgi?acc=GSE73731. Data obtained from TCGA database is available at: https://portal.gdc.cancer.gov/.

## Author Contributions

HY and YW conceived, designed, and conducted the study, as well as wrote the manuscript. ZZ selected and pre-processed the data. All authors reviewed the manuscript and participated in the language modification.

## Conflict of Interest

The authors declare that the research was conducted in the absence of any commercial or financial relationships that could be construed as a potential conflict of interest.

## Footnotes

1 https://www.ncbi.nlm.nih.gov/geo/

2 https://portal.gdc.cancer.gov/
